# Predictive Biomarkers for Checkpoint Inhibitor-Based Immunotherapy in Hepatocellular Carcinoma: Where Do We Stand?

**DOI:** 10.3389/fonc.2021.803133

**Published:** 2021-12-17

**Authors:** Alessandro Rizzo, Angela Dalia Ricci, Alessandro Di Federico, Giorgio Frega, Andrea Palloni, Simona Tavolari, Giovanni Brandi

**Affiliations:** Medical Oncology, IRCCS Azienda Ospedaliero-Universitaria di Bologna, Bologna, Italy

**Keywords:** hepatocellular carcinoma, HCC, immunotherapy, biomarkers, PD-L1

## Abstract

Hepatocellular carcinoma (HCC) remains the sixth most commonly diagnosed malignancy worldwide, still representing an important cause of cancer-related death. Over the next few years, novel systemic treatment options have emerged. Among these, immune checkpoint inhibitors (ICIs) have been widely evaluated and are under assessment, as monotherapy or in combination with other anticancer agents in treatment-naïve and previously treated patients. In particular, the approval of the PD-L1 inhibitor atezolizumab plus the antiangiogenic agent bevacizumab as front-line treatment for advanced HCC has led to the adoption of this combination in this setting, and the IMbrave 150 phase III trial has established a novel standard of care. However, several questions remain unanswered, including the identification of reliable predictors of response to ICIs in HCC patients. In the current paper, we will provide an updated overview of potentially useful predictive biomarkers of response to immunotherapy in advanced HCC. A literature search was conducted in September 2021 of Pubmed/Medline, Cochrane library and Scopus databases.

## Introduction

Hepatocellular carcinoma (HCC) remains a lethal tumor with a 5-year survival rate of less than 20% for patients at all stages, accounting for more than 75% of all primary liver malignancies and resulting in 83 million deaths in 2020 (1, 2). A number of liver diseases has been historically associated with the onset of HCC, including liver cirrhosis, hepatitis virus infection, and non-alcoholic liver disease (NAFLD), with some of these etiologies that have been suggested to play a role in modifying responses to systemic treatments (3, 4). Potentially curative treatment options for patients with limited disease [encompassing early-stage Barcelona Clinic Liver Cancer (BCLC) 0/A and intermediate-stage BCLC B HCC] include radical surgery, liver transplantation, and locoregional approaches such as chemoembolization and radiofrequency ablation, while BCLC C patients usually receive systemic therapies (5–9). We have recently witnessed the emerging and advent of immune checkpoint inhibitors (ICIs) in a wide number of hematological and solid tumors, and these agents have caused unprecedented treatment paradigm shifts in a relatively short period of time (10–12). ICIs are able to contrast immune checkpoint-related molecules - including programmed cell death-1 (PD-1), cytotoxic T-lymphocyte-associated antigen 4 (CTLA-4), lymphocyte-activation gene 3 (LAG-3) (13, 14) – and these agents have been recently tested and are under evaluation in HCC patients. While results of ICI monotherapy have been disappointing so far (15–17), the recently presented and published phase III IMbrave150 study comparing the PD-L1 inhibitor atezolizumab plus bevacizumab versus sorafenib monotherapy as front-line treatment approach for advanced HCC patients has established a new standard of care (18–20). In particular, patients receiving the atezolizumab plus bevacizumab combination showed impressive benefits in terms of several endpoints, such as progression-free survival (PFS), overall survival (OS), objective response rate (ORR) and complete response (CR) rate, as also confirmed by the updated results presented by Finn and colleagues reporting a median OS of 19.2 months in the experimental arm (21, 22). These results are destined to mark a new era in this setting, and ICIs seem to have found their role in advanced HCC patients in combinatorial strategies (23); however, the use of immunotherapy in HCC has been associated with several limitations in this setting (24). In particular, the identification of predictive biomarkers is urgently needed to guide the therapeutic process since approximately half of advanced HCC patients treated with ICIs do not benefit from this therapeutic approach (25–27). Thus, a detailed discussion and comprehension of this issue is of pivotal importance, and several potential predictors such as programmed death ligand 1 (PD-L1), tumor mutational burden (TMB), gut microbiota and several others are under assessment (28–30). We performed a research on Pubmed/Medline, Cochrane library and Scopus using the following keywords “HCC” OR “hepatocellular carcinoma” OR “liver cancer” AND “PD-L1” OR “TMB” OR “MSI” OR “MMR” OR “DDR” OR “gut microbiome” OR “predictive biomarkers” OR “predictors of response” AND “pembrolizumab” OR “PD-L1” OR “immune checkpoint inhibitors” OR “immunotherapy” OR “PD-1 inhibitor” OR “atezolizumab” OR “durvalumab” OR “tremelimumab” OR “nivolumab”.

## PD-L1

As a matter of fact, we have recently witnessed the approval of several ICIs according to PD-L1 expression in different malignancies, as in the case of the PD-1 inhibitor pembrolizumab in previously untreated patients with advanced non-small cell lung cancer (NSCLC) with PD-L1 Tumor Proportion Score (TPS) of 50% or more (31–33). However, PD-L1 assessment presents several issues, such as the use of different antibody clones as well as distinct cut-off thresholds (34, 35); in addition, PD-L1 is determined on tumor cells or immune infiltrating cells, something that further limits the standardization of this biomarker across different tumor types (36, 37).

As regards the field of HCC, around 10% of tumor cells have been suggested to express PD-L1, with this biomarker reporting a putative prognostic value (38); in particular, PD-L1 positive HCCs have been associated with shorter survival and recurrence-free survival, with recent retrospective trials reporting a possible link between macrovascular invasion, poorly differentiated or undifferentiated disease, high serum alfa-fetoprotein (AFP), and PD-L1 expression (39). In terms of predictive value, few data are available so far regarding the role of PD-L1 status in advanced HCC patients receiving ICIs as monotherapy or part of combinatorial strategies; in addition, not only these data are sparse, but are also conflicting. For example, the phase III CheckMate 459 trial comparing nivolumab monotherapy versus sorafenib as front-line treatment showed higher response rate in PD-L1 positive advanced HCCs treated with immunotherapy (40); similarly, the KEYNOTE-224 study on pembrolizumab observed that PD-L1 positive expression was associated with improved response in patients receiving ICI monotherapy (41). On the other hand, in the phase I/II CheckMate 040 study including 174 advanced HCCs with available PD-L1 status, a non-statistically significant difference was reported between PD-L1 positive and PD-L1 negative patients treated with nivolumab (26% versus 19% in PD-L1 positive and PD-L1 negative HCCs, respectively, by using a cut-off value of 1% of tumor cells) (42). These controversial results observed across different trials on ICIs are not particularly surprising since all these studies were not adequately powered to differentiate responders and non-responders according to PD-L1 expression. In addition, and similar to other solid tumors, the current lack of validated and standardized methodologies and cut-off values clearly evaluating PD-L1 status further complicates this scenario, also considering the dynamic nature of PD-L1 due to its high spatial and temporal heterogeneity (43–45). Lastly, responses to ICIs have been observed in PD-L1 positive and PD-L1 negative patients, with this evidence calling into question the effective utility of this biomarker in this setting.

## TMB and MMR/MSI

The term tumor mutational burden (TMB) is commonly used to quantify the number of nonsynonymous mutations observed in the tumoral genome (46, 47), and tumors with higher number of mutations have been associated with the production of higher levels of neoantigens and enhanced immune response (48, 49). Recent years have witnessed the presentation and publication of several trials specifically focused on the association between high TMB and response to immunotherapy, with the former suggested to be a predictive biomarker in this setting (50, 51). However, TMB has not been validated so far and its use remains investigational.

**Figure 1:**
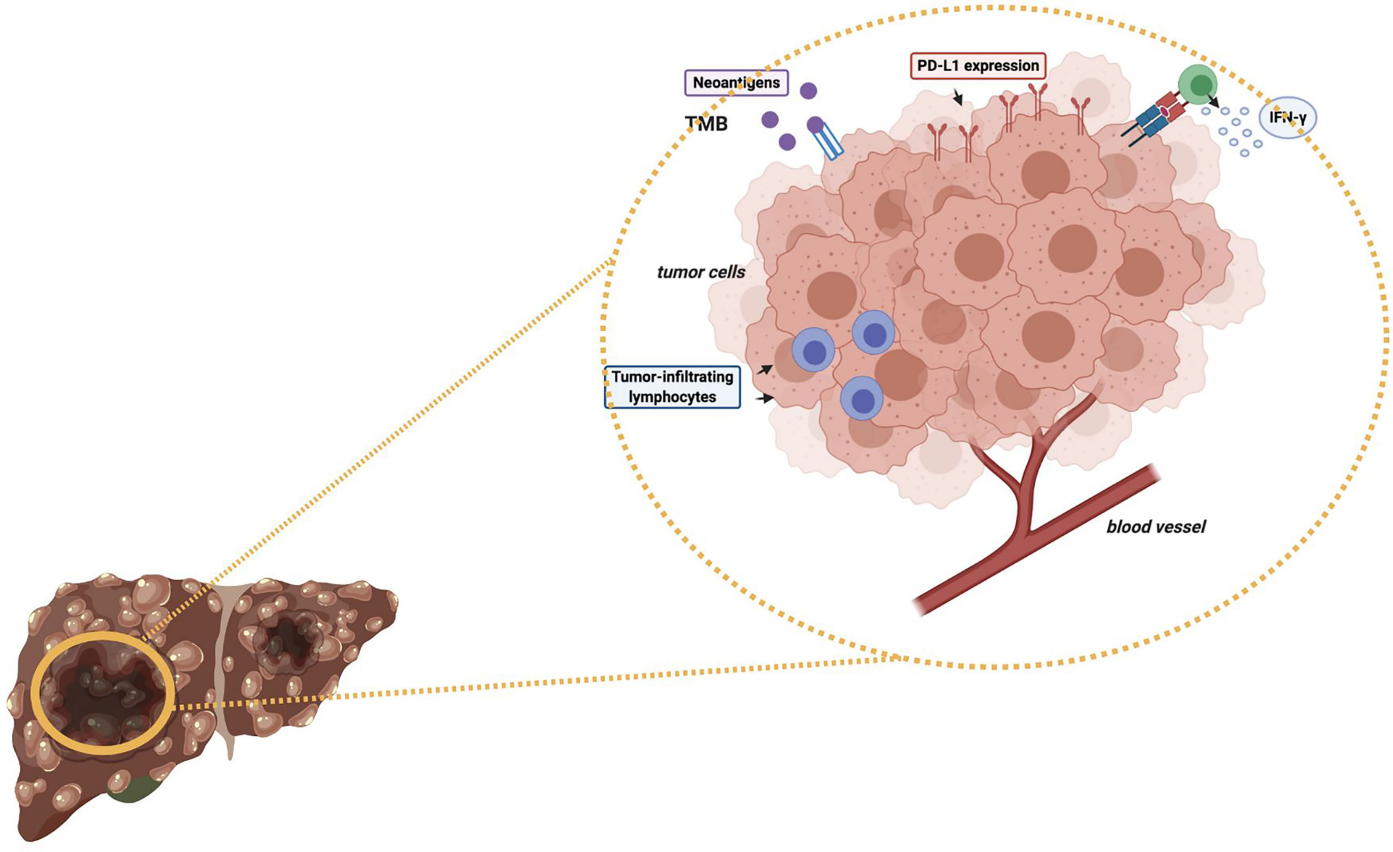
Schematic figure representing some potential predictive biomarkers of response to immunotherapy in HCC patients. *TMB, Tumor Mutational Burden; PD-L1, Programmed Death-Ligand 1; IFN-γ, Interferon-gamma*.

As regards immunotherapy in HCC, the role of high TMB in modifying and determining response to ICIs as monotherapy or in combination with other anticancer agents remains to be elucidated (**Figure 1**) (47, 52). Of note, some recent studies have reported a median of 4-5 mutations per megabase in HCC patients (53); in particular, Ang and colleagues recently investigated the prevalence of TMB across 755 HCC patients, and only 0.8% of included subjects presented high TMB, with the same trial reporting no association between high TMB and response to ICIs (54). Another interesting research avenue regards the suggested differences in terms of TMB across geographical areas and according to ethnicity; in fact, in a study by Tang and colleagues the authors reported that Chinese patients with HCC presented higher median TMB compared with patients from Western areas (9.3% and 1%, respectively) (55). Another recent study by Wong et al. performed targeted Next Generation Sequencing (NGS) on archival and fresh samples of 29 HCCs (56); of note, the trial highlighted lower TMB in fresh samples, with a median of 958.39 mutations per megabase in archival samples versus 2.51 mutations per megabase in fresh samples. Therefore, the authors suggested that fresh HCC samples could represent a better source of tumor DNA compared with archival samples (56).

Another potentially meaningful predictor under assessment in cancer immunotherapy is microsatellite instability (MSI) status, as witnessed by landmark studies published over the last decade (57, 58). In fact, several trials have reported that deficiencies in DNA mismatch repair (MMR) may cause a “hypermutator” phenotype commonly defined as microsatellite instability (MSI) (59, 60); in particular, loss of mutations in MMR-related genes including MLH1, MSH2, MSH6, and PMS2 impair the MMR system (61, 62). In addition, high mutation rates in somatic cells have been suggested to boost the neoantigen load, resulting in lymphocyte activation and consequent increase of the efficacy of cancer immunotherapy. Despite the use of MSI has entered into the clinical practice leading to the use of ICIs as first-line (e.g., colorectal cancer) or in second- and later-line, available literature regarding the role of MSI as a predictive biomarker in HCC patients receiving ICIs is mainly based on case reports (63). Of note, MSI-H is deemed to be quite a rare finding, with retrospective studies reporting a proportion of approximately 3% of cases (64, 65). In the previously cited study, Ang and colleagues highlighted that only 0.2% of included HCC patients were MSI-H and had high TMB, suggesting that “hypermutated phenotypes” seem to be a rare evidence in this primary liver tumor (54). The role of MSI-H in this setting remains to be fully elucidated.

## Gene Mutations

The advent of molecular profiling has produced notable paradigm shifts in the management of several hematological and solid tumors (57, 66). Of note, not only several genetic mutations have been suggested to represent a therapeutic target and to identify “oncogene-addicted disease”, but some of these aberrations have been also associated with response to ICIs (67, 68).

As regards HCC, a recent study by Harding et al. performed NGS to evaluate the association between systemic treatments (such as PD-1 inhibitors, CTLA-4 inhibitors, anti-LAG3, etc.) and genetic aberrations including Wnt/β-catenin mutations (69). Interestingly, in 31 patients with advanced HCC receiving heterogeneous schedules and types of ICIs, Wnt/β-catenin mutated HCCs reported a statistically significant association with resistance to immunotherapy and shorter median OS and PFS compared with wild-type patients, with a median OS of 9.1 and 15.2 months, and a median PFS of 2.0 months and 7.4 months, respectively (69). In addition, around half of wild-type HCCs reported a response to ICIs, while no responses were reported in patients with Wnt/β-catenin mutations (69). This evidence has been also confirmed by other trials on this topic, with malignancies harboring β-catenin aberrations reporting a putative resistance to immunotherapy in mouse models (70, 71).

Among the most commonly highlighted genetic mutations in HCC, *TP53* gene mutations are mutually exclusive with *CTNBB1* mutations and HCCs with these aberrations have been suggested to have specific features in terms of tumor microenvironment (TME) (72); in fact, according to recent studies these malignancies report higher Foxp3^+^ Treg infiltration and lower CD8^+^ T cell infiltration (73). At the same time, it is worth noting that these results remain preliminary, with other trials reporting better clinical outcomes and higher tumor-infiltrating lymphocytes (TILs) in HCC patients with TP53 mutations (74, 75).

DNA damage repair (DDR) gene alterations have recently emerged as potential predictive biomarkers of response to immunotherapy due to the genomic instability associated with these aberrations (76, 77). In fact, alterations in DDR genes have been involved in the impairment of DNA repair processes, hesitating in accumulation of DNA damages due to genomic stability loss (78, 79). Despite several preclinical trials have highlighted a synergistic effect for *PARP* inhibitors plus immunotherapy in cancer patients with these aberrations, no data are available regarding this approach in advanced HCC so far (80, 81).

Immune-related long noncoding RNAs (lncRNAs) represents another emerging line of research in the field of predictors of response to immunotherapy (82, 83). Interestingly, a trial by Peng et al. suggested an association between PD-L1 and CTLA-4 with the lncRNA MIR155 host gene, and this gene seems to appear as a promising predictive biomarker of response to ICIs (84). Moreover, recent trials have highlighted that HCC patients with specific immune-related lncRNAs signatures may present enhanced immune cell infiltration and high PD-L1, PD-L2, and IDO1 expression (85). Despite the use of lncRNAs seems promising, the application of these potential predictors remains far from everyday clinical practice.

## Gut Microbiota

Several novel focuses of research are under active development, including gut microbiome (86–89); for example, Zheng et al. recently suggested dynamic changes occurring in the gut microbiome during immunotherapy in HCC patients (90). In this interesting report, fecal samples from patients responding to ICIS showed higher taxa richness compared to fecal samples of non-responders, suggesting that human microbiota could have a notable impact on responses to immunotherapy in HCC patients (90). Despite the application of gut microbiota in modulating response to immunotherapy remains far from clinical practice, the landmark role of gut microbiota in modulating innate and adaptive immunity supports makes this research avenue particularly promising (91–96).

## Future Perspectives

Recent years have seen the advent of immunotherapy, and the identification of specific histological and molecular predictors of response to ICIs represents one of the current and future challenges in several tumor types, including HCC (97). In fact, only a proportion of HCC patients seems to benefit from immunotherapy, highlighting the need for a deeper understanding of predictors of response. In the near future, the HCC medical community is called to more efforts aimed at evaluating novel biomarkers of response to ICIs, considering tumor-intrinsic (e.g., PD-L1 expression, TMB, MSI status, etc.), immune-specific, and combinatorial biomarkers. In fact, a combination of PD-L1 with other biomarkers would probably be more impactful compared to a single predictor of response.

The identification of predictive biomarkers of response to immunotherapy remains a priority in HCC, especially considering that the number of indications and patients receiving ICIs is supposed to further increase in the near future. Thus, the question whether PD-L1 expression, TMB and other potential predictors could be applied to select the right patients to receive immunotherapy and to provide useful information for disease-monitoring and treatment-decision making remains critical. Further investigations are warranted in this direction.

## Author Contributions

All authors contributed to the article and approved the submitted version.

## Conflict of Interest

The authors declare that the research was conducted in the absence of any commercial or financial relationships that could be construed as a potential conflict of interest.

## Publisher’s Note

All claims expressed in this article are solely those of the authors and do not necessarily represent those of their affiliated organizations, or those of the publisher, the editors and the reviewers. Any product that may be evaluated in this article, or claim that may be made by its manufacturer, is not guaranteed or endorsed by the publisher.
